# Comparative Secretome Analyses of *Mycoplasma bovis* Virulent and Attenuated Strains Revealed MbovP0145 as a Promising Diagnostic Biomarker

**DOI:** 10.3389/fvets.2021.666769

**Published:** 2021-06-18

**Authors:** Hui Zhang, Guyue Hu, Doukun Lu, Gang Zhao, Yiqiu Zhang, Muhammad Zubair, Yingyu Chen, Changmin Hu, Xi Chen, Jianguo Chen, Huanchun Chen, Liguo Yang, Aizhen Guo

**Affiliations:** ^1^The State Key Laboratory of Agricultural Microbiology, Huazhong Agricultural University, Wuhan, China; ^2^College of Veterinary Medicine, Huazhong Agricultural University, Wuhan, China; ^3^Key Laboratory of Development of Veterinary Diagnostic Products, China Ministry of Agriculture and Rural Affairs, Huazhong Agricultural University, Wuhan, China; ^4^Hubei International Scientific and Technological Cooperation Base of Veterinary Epidemiology, Huazhong Agricultural University, Wuhan, China; ^5^Key Laboratory of Ruminant Bio-Products of Ministry of China Agriculture and Rural Affairs, Huazhong Agricultural University, Wuhan, China; ^6^College of Animal Science and Technology, Huazhong Agricultural University, Wuhan, China

**Keywords:** *Mycoplasma bovis*, secretome, proteomics, virulence, biomarker, cattle

## Abstract

Mycoplasmas are successful pathogens both in humans as well as in animals. In cattle, *Mycoplasma bovis* (*M. bovis*) is known to be responsible for serious health complications, including pneumonia, mastitis, and arthritis. However, *M. bovis* pathogenesis remains unclear. Secreted proteins of *M. bovis* could influence infection and modify host defense signaling pathways after they enter their extracellular space in the host micro-environment. Therefore, this study was aimed to compare the secretomes of *M. bovis* HB0801 virulent (P1) and attenuated (P150) strains and identify potential pathogenesis-related secreted proteins and biomarkers. The cells of P1 and P150 strains were grown in pleuropneumonia-like organism medium to log phase and then transferred to phosphate-buffered saline for 2 h. Then, the supernatant was analyzed by using label-free quantitative proteomics, and 477 potential secreted proteins were identified. Combined with the bioinformatics prediction, we found that 178 proteins were commonly secreted by the P1 and P150 strains, and 49 of them were encoded by mycoplasmal core genes. Additionally, 79 proteins were found to have a different abundance between the P1 and P150 strains. Among these proteins, 34 were more abundant and uniquely expressed in P1, indicating a possible association with the virulence of *M. bovis*. Three differentially secreted proteins, MbovP0145, MbovP0725, and MbovP0174, as well as one equally secreted protein, MbovP0481, as positive control and one protein of inner membrane, MbovP0310, as negative control were, respectively, cloned, expressed, and evaluated for antigenicity, subcellular location, and the secretion nature with their mouse antisera by western blotting and colony immunoblotting assay. Among them, MbovP0145 was confirmed to be more secreted by P1 than P150 strain, highly reactive with the antisera from naturally infected and P1 experimentally infected cattle but not with the P150 vaccinated calves, indicating its potential as a diagnostic antigen. In conclusion, these findings may represent the most extensive compilation of potentially secreted proteins in mycoplasma species and the largest number of differentially secreted proteins between the virulent and attenuated *M. bovis* strains to date and provide new insights into *M. bovis* pathogenesis and diagnosis.

## Introduction

Mycoplasma species that cause significant and important diseases in humans and animals are characterized by their small genomes (0.58–2.2 Mb) and lack of cell walls (1). They are often host specific, and most are highly pathogenic (1). In cattle, infection by *Mycoplasma bovis* can manifest as pneumonia and a plethora of other clinical signs, including mastitis, arthritis, keratoconjunctivitis, meningitis, otitis media, and genital tract diseases that are likely to result in infertility and abortion (2–4). *M. bovis* pneumonia is a major contributor to the bovine respiratory disease complex (5), a worldwide concern that has a significant detrimental economic impact on the cattle industry. *M. bovis* pneumonia was first reported in China in 2008 (6) and later became an epidemic throughout the whole country. At that time, 97.7% Chinese isolates were identified as the same genotype ST-10 by multilocus sequence typing (7). However, the current control strategies for *M. bovis* based on chemotherapy are poorly effective due to the organism's innate resistance to β-lactam antibiotics and its rapidly acquired resistance to other antibiotics (8). Therefore, alternative strategies, such as the development of novel vaccines, are urgently needed to control this disease. However, vaccine development is hindered by the poor understanding of the virulence-related factors and immunogenicity of *M. bovis*.

Previously, the *M. bovis* HB0801 strain (P1) isolated in China from the lung tissue of a cattle with *M. bovis* pneumonia was continuously passaged *in vitro* 180 times, and the virulence and immunogenicity of the passaged P115, P150, and P180 strains were evaluated in cattle. The attenuated P150 strain was ultimately selected as a live vaccine strain (9). Comparative genomic analyses demonstrated that the P150 strain shares a highly similar genome structure and coding DNA sequences with P1, except for a 14.2-kb fragment that is missing 14 putative genes and 46 non-sense single-nucleotide polymorphisms (10, 11). A calf experiment demonstrated that inoculation of the P150 strain can provide good protection from a P1 challenge (11, 12). Therefore, the differentially abundant proteins between P1 and P150 strains were hypothesized to be responsible for the pathogenicity and immunogenicity of *M. bovis*.

The proteins secreted by bacterial pathogens are well-known to influence infection and to modify host defense signaling pathways upon their entry into the extracellular space in the host micro-environment. These proteins are largely toxins, enzymatic effectors, and antigens secreted by types I–VII secretion apparatuses (12) and are therefore considered to represent useful targets for the development of new vaccines and drugs. However, the progress in identifying the proteins secreted by mycoplasmas has lagged in the past because mycoplasma species lack typical secretion systems. Recently, an involvement of some soluble proteins secreted by mycoplasmas has been proposed in basic metabolic processes and pathogenesis. For example, a membrane protein P80 was a secreted protein of *Mycoplasma hominis* released by its N-terminal region to recognize a signal peptidase (13). Similarly, a nuclease MbovP580 from *M. bovis* was identified as a secreted protein associated with cytotoxicity (14). The secretion of several serine protease and peptidase with caseinolytic activity has also been reported for the ruminant mycoplasmas *Mycoplasma mycoides* subsp. *capri* and *Mycoplasma bovirhinis MV5* (15). Further characterization of mycoplasma secretomes could therefore provide a better avenue for the exploration of the interactions occurring between mycoplasmas and their hosts.

A proteomic analysis of extracellular proteins *via* two-dimensional (2-DE) electrophoresis was first reported for *Mycoplasma synoviae*, and 27 proteins were found in a protein-free medium (16). Similarly, a total of 111 proteins were identified in the secretome of *Mycoplasma capricolum*, and a further three key secreted proteins were confirmed as prominent in an acidic pH medium (17). A comparative analysis between *Mycoplasma hyopneumoniae* and *Mycoplasma flocculare* revealed several secreted proteins (18). Interestingly, a vaccine with a cocktail formulation that included seven secreted proteins of *M. hyopneumoniae* was reported to enhance the immune response (19). Due to technical limitation, previous analyses of mycoplasma secretomes were carried out mainly by one- or two-dimensional electrophoresis and mass spectrometric analysis. In the recent years, an alternative approach for the identification of large numbers of proteins is the label-free liquid chromatography–tandem mass spectrometry (LC–MS/MS) method (20). This is a high-throughput quantitative proteomic technique that allows straightforward processing of samples and the successful identification of novel biomarkers. The especially high sensitivity enables the detection of low levels of proteins.

In the present study, a systemic secretome comparison was performed based on bioinformatics analysis coupled with label-free LC–MS/MS between the P1 and P150 strains of *M. bovis*. The objectives were to characterize the *M. bovis* secretome, to identify key molecules associated with the pathogenicity of *M. bovis*, and to provide target candidates for the development of novel diagnostic agents and vaccines.

## Materials and Methods

### Culture of Bacterial Strains

*M. bovis* HB0801 wild-type strain (P1) (GenBank accession no. NC_018077.1) was isolated from the lung of a diseased cattle in Hubei Province, China (21). Its attenuated strain, *M. bovis* P150 strain (P150) (GenBank accession no. CP007590.1), was derived from P1 after continuous passaging *in vitro* 150 times and was maintained in our laboratory (9). In addition, 11 clinical isolates of *M. bovis* were recovered during 2008–2013 from the nasopharynges, lungs, and joints of feedlot cattle and kept in our laboratory. All strains were routinely propagated in pleuropneumonia-like organism (PPLO) medium supplemented with 10% horse serum (Hyclone, South Logan, USA) and incubated at 37°C in 5% CO_2_.

### Serum Samples From Experimentally and Naturally Infected Cattle

A total of 178 serum samples were collected from 60 experimentally infected calves, 40 naturally infected calves, and 78 uninfected calves as determined by isolations conducted in our laboratory. The four types of serum samples from the calves' post-immunization and post-challenge were as follows:

(i) A total of 60 serum samples from experimentally infected calves were used for the assessment of safety and efficacy of the vaccine strain. **Sixty** calves were inoculated intratracheally with *M. bovis* P1 at a dose of 10^9^ cfu/calf. At 60 days post-infection, the animals were euthanized for necropsy, and the antibody titers of serum samples were measured with a commercial ELISA kit (#BIO K 302, Bio-X Diagnostics S.A., Brussels, Belgium) as described previously (9, 22).

(ii) A total of 40 serum samples were obtained from naturally infected calves from 37 feedlots in Hubei, China. The disease was confirmed by culture isolation and identification in our laboratory (23).

(iii) A total of four serum samples were tested from calves post-P150 immunization and post-P1 challenge. Four 2-month-old, weaned calves were intranasally inoculated with P150 [1 × 10^8^ colony-forming units (CFU)/calf] on day 0. On day 35, each calf was then intratracheally challenged with P1 (1 × 10^9^ CFU/calf). Based on the days post-immunization (dpi) with the P150 strain, serum samples were collected at 0, 7, 14, 21, 28, 35, 42, 49, 56, 63, and 70 dpi from all calves (11).

(iv) Serum samples were also obtained from 78 locally bred beef calves, 5–6 months of ages, as negative controls, as confirmed by nasal swab cultures and PCR detection. The serum titers were determined with a commercial ELISA kit (#BIO K 302, Bio-X Diagnostics S.A., Brussels, Belgium).

### Ethics Approval and Consent to Participate

The protocol (HZAUMO-2018-027) for animal experiments was approved by the Committee on the Ethics of Animal Experiments at Huazhong Agricultural University, and all experiments were conducted in strict accordance with the Guide for the Care and Use of Laboratory Animals, Hubei Province, China.

### Prediction of Secreted Proteins With Bioinformatic Tools

The coding gene sequences of *M. bovis* HB0801 were downloaded from the NCBI database (https://www.ncbi.nlm.nih.gov/). The putative signal peptides were predicted using SignaIP 5.0 (http://www.cbs.dtu.dk/services/SignalP/) and Predisi (http://www.predisi.de/), and the lipoprotein motifs were predicted in the first 70 amino acids of each sequence using LipoP 1.0 (http://www.cbs.dtu.dk/services/LipoP/) to remove cytoplasmic prediction proteins. The non-classical secreted proteins were predicted using SecretomeP 2.0 (http://www.cbs.dtu.dk/services/SecretomeP/) by selecting the default options for bacteria and the recommended N-N scores ≥0.5 as the threshold for the resultant proteins. All the secreted proteins determined by each predictor were then merged together, and the resultant list was scanned using TMHMM 2.0 (http://www.cbs.dtu.dk/services/TMHMM/) to be devoid of any transmembrane helices. The remaining proteins with ≥1 transmembrane motifs were further analyzed with Phobius (http://phobius.sbc.sue/) to identify possible α-helical conformations in the N-terminal region of the proteins that belong to a signal sequence but could be mistakenly classified as a transmembrane region. Any analyzed proteins predicted to have a signal sequence were added to the list of secreted proteins. The proteins predicted using the bioinformatics pipeline were defined as the secreted proteins.

The secretion type of each protein identified was categorized as either “classical,” if a signal peptide was identified by at least two software programs, or “non-classical” if the SecretomeP prediction was positive but no signal peptide was identified. If no signal peptide was predicted and the SecretomeP analysis was negative, the protein was assigned to the “undefined” secretion type. The subcellular localizations of the proteins were predicted by using PSORTb version 3.0.2 (http://www.psort.org/psortb/).

### Sample Preparation for Label-Free Quantitative Proteomic Analysis

The *M. bovis* strains P1 and P150 were cultivated in complete PPLO broth with 10% horse serum to the log phase at 37°C and 5% CO_2_ for 18 h. Then, the cells were collected by centrifugation (15,000 *g*, 20 min, 4°C), suspended in the same volume of sterile phosphate-buffered saline, and incubated at 37°C for 2 h. The viability of the *M. bovis* strains was determined by plating serial dilutions of liquid cultures onto PPLO agar plates every 30 min. Then, the cells were centrifuged at 15,000 *g* for 20 min at 4°C, and the supernatant was collected and passed through a 0.22-μm filter to remove possible big cell clusters and debris. The final supernatant containing the secreted products was concentrated to 80 μl by ultrafiltration to remove molecules smaller than 5 kDa. In the concentrated samples, 100 μl SDT lysis buffer (4% SDS, 1 mM DTT, and 100 mM Tris-HCl, pH 7.6) was added, and then the concentrated secreted products were stored at −80°C until use. A bicinchoninic acid (BCA) protein assay kit (Beyotime Biotechnology, China) was used for protein quantification, and 12% SDS-PAGE and *M. bovis* plate counting assays were used to control the preparation quality. The secretome samples were expected to differ in appearance from the *M. bovis* pellets, and the ratio of live *M. bovis* number in the final preparation to the original cell number in the culture was required to be less than five cells per 10^8^ cells.

About 100 μg of each protein preparation was mixed with 10 mM DTT and placed in a boiling water bath for 5 min, followed by incubation with 50 mM iodoacetamide at room temperature (RT) in the dark for 30 min. The samples were then digested by adding 40 μl trypsin buffer (4 μg trypsin in 40 μl 100 mM NH_4_HCO_3_) and incubating at 37°C for 16–18 h. The digestion step was repeated twice. The samples were then desalted on a reverse phase (RP) trap column (C18 Cartridge, Agilent Technologies, Wilmington, DE) and then combined with 40 μl 0.1% formic acid, lyophilized under vacuum, and stored at −80°C until further analysis. Three independent replicates were prepared for each *M. bovis* strain.

The peptide mixtures generated for each sample were used for protein identification using a nano-LC–MS/MS system consisting of a nano-HPLC system (EASY-nLc1000) and a linear trap quadrupole mass spectrometer. Mobile phase A consisted of HPLC-grade water containing 0.1% formic acid, while phase B consisted of 84% HPLC-grade acetonitrile containing 0.1% formic acid. For analytical separation, the flow rate was 400 nl/min using the following linear gradient: phase B (gradient for 2 h in total): 0–55% for 110 min, 55–100% for 5 min, and 100% for 5 min. The eluent was then introduced into the Q-exactive mass spectrometer with the ESI spray voltage set at 3.2 kV. For MS survey scanning, each cycle consisted of one full MS scan and 20 MS/MS scans of the peptide fragments.

### Protein Identification and Quantification

The MS/MS data were analyzed using MaxQuant (version 1.3.05) against Mbov_HB0801 (P17188_HB0801_amino_acids_762_20170502.fasta, a self-built database). The search parameters included a trypsin digestion and two missed cleavages of trypsin, with allowance for an error of 6 ppm for the full MS or 20 ppm for the MS/MS spectra study. The carbamidomethylation of cysteine was specified as a fixed modification, whereas the oxidation of methionine and the acetylation of the N-terminal ends of proteins were specified as variable modifications.

Proteins were quantified using razor and unique peptides. The false discovery rate (FDR) for the proteins and peptides was calculated and required to be ≤ 0.01. The final step was an automatic matching of each peptide within a mass and time tolerance window (0.5 Da and 2 min, respectively). Only proteins identified by at least one unique peptide and a minimum of two valid values in at least one group were considered, while the proteins excluded were identified only by site or were marked as contaminants. A protein with at least two LFQ intensity values showing *p* 0.05 and log_2_(fold change) >2 was considered to show a statistically significant difference.

### Identification of Overlapped Proteins Between Genome Prediction and Proteomics Analysis

The principle of “the greatest common divisor” was used to identify overlapped proteins between the genome prediction and the label-free proteomic results. The function of each identified protein was annotated by blasting based on the eggnog-mapper (http://eggnog-mapper.embl.de/). The subcellular localizations and secretion types were analyzed for the overlapped proteins between P1 and P150. These proteins were also mapped to Gene Ontology (GO) terms using Blast2GO, and the GO enrichment was analyzed using the tool of OmicShare, a free online platform for data analysis (www.omicshare.com/tools). The proteins associated with virulence were identified using the VirulentPred web server to blast on the basis of a similarity search with a threshold value ≥0 (http://203.92.44.117/virulent/index.html).

### Verification of Predicted Secreted Proteins

Five proteins were selected to verify their secretion and differential expression between P1 and P150 strains. These included three differentially secreted proteins: MbovP0145 and MbovP0725, which had a higher abundance in P1, and MbovP0174, which had a higher abundance in P150. MbovP0481, which had equal abundance in both strains, was included as a positive control and was previously reported to be a secreted protein released through extracellular vesicles (EVs) (24). MbovP0310 (SecA-like protein), which is a marker protein of the inner membrane in *Escherichia coli*, was included as a negative control.

The genes were first commercially synthesized by mutating the TGA tryptophan codon of *M. bovis* into TGG (tryptophan codons in *E. coli*) and cloning into the pET-30a (+) vector using corresponding restriction enzymes. The recombinant plasmids were then transformed into competent *E. coli* BL21 cells. The expression of the recombinant proteins and solubility tests were performed as previously described (14). The purity of the recombinant proteins was analyzed by 12% SDS-PAGE, and the protein concentrations were determined using the BCA protein assay kit. In addition, the protein sequence of *M. bovis* MbovP0145 and its orthologs of other mycoplasma species were retrieved from NCBI databases. The alignment of amino acid sequences was performed with BLASTP, while multiple alignments were analyzed by DNAstar software.

To confirm the conservation of MbovP0145, western blotting assay was performed to check MbovP0145 expression in different clinical strains as well as in P1 and P150 strains. Then, a 20-μg sample of the whole cell protein from 11 *M. bovis* strains was transferred onto polyvinylidene difluoride (PVDF) membrane. Mouse anti-rMbovP0145 polyclonal antibodies and HRP-conjugated goat anti-mouse IgG (SouthernBiothech, Birmingham, USA) were used as primary and secondary antibodies, respectively. *M. bovis* PG45 was used as the control.

### Production of Polyclonal Antibodies

Mouse polyclonal antisera against five selected recombinant proteins were generated in 4-week-old female BALB/c mice. The mice were immunized through a subcutaneous injection with 100 μg of each recombinant protein emulsified in Freund's complete adjuvant for the priming or Freund's incomplete adjuvant for the boosters (Sigma, St Louis, MO, USA). The immunization was conducted three times at a 2-week interval. The serum titers were assessed by ELISA as previously described (14). Once a peak titer was achieved, the mice were euthanized, and their antisera were collected and stored at −20°C until use.

### Immunoblotting Analysis

Western blotting was performed to determine the locations of the recombinant proteins. The whole cell proteins (WCP) and secretomes of *M. bovis* strains P1 and P150 were extracted as detailed above, separated by 12% SDS-PAGE, and transferred to PVDF membranes. After blocking, the signals were probed with mouse polyclonal antisera to specific recombinant proteins (diluted 1:200), and immunoblots were developed with HRP-conjugated goat anti-mouse IgG (1:3,000, SouthernBiotech, Birmingham, AL, USA) for 1 h.

Colony immunoblotting analysis was also performed to detect *M. bovis* cell surface or extracellular proteins, as described previously (25). *M. bovis* colonies grown on agar plates were transferred onto PVDF membranes through close contact. After blocking with 5% skim milk, the membranes were incubated with mouse antiserum (1:200) against each protein at RT for 2 h and then incubated with HRP-conjugated goat anti-mouse IgG antibodies. Both the western and colony blots were developed using an enhanced chemiluminescence substrate kit (Advansta, California, USA).

### Antigenicity Verification of the Selected Proteins

The antigenicity of selected proteins was assessed by western blotting and ELISA using cattle sera previously collected from naturally infected, experimentally P1-infected, and uninfected cattle as described above. The western blot assay used the pooled positive sera (1:200) from 15 experimentally infected calves and pooled negative sera from 15 uninfected calves as the primary antibody. Further reactivity of the recombinant proteins was detected using individual cattle serum with an indirect ELISA (iELISA), established as described below. Briefly, 100 μl of each purified recombinant protein was coated onto the wells of an ELISA plate (100 ng/well) and reacted with the serum samples of the 15 infected and 15 uninfected cattle.

### Establishment of rMbovP0145-Based iELISA

An indirect rMbovP0145-based ELISA was established as described previously (22). Each well of 96-well plates was coated with the recombinant rMbovP0145 protein (100 ng/well) and blocked with 5% skimmed milk powder in PBS containing 0.05% Tween 20 (PBST). The positive and negative cattle sera described above and the testing sera described below were diluted to 1:100 with PBS. HRP-conjugated goat anti-bovine IgG (H+L) was pre-diluted for use at 1:5,000 in PBS. After the final wash, the 3,3′,5,5-tetramethylbenzidine (TMB)/H_2_O_2_ substrate was added (100 μl/well), and the plates were incubated for 10 min at RT. The reaction was stopped by addition of 0.2 M H_2_SO_4_ (50 μl/well), and the absorbance was measured at 450_nm_ with a hybrid multimode microplate reader (Envision, PerkinElmer, USA). The sample-to-positive (S/P) values were calculated using the following formula: S/P = (OD_sample_ - OD_negativecontrol_)/(OD_positivecontrol_ - OD_negativecontrol_). The cutoff of S/P for discrimination of sera reactivity was determined by receiver operating characteristic (ROC) curve as previously described (26). The testing serum samples with known background were previously collected from 78 uninfected calves and 100 calves infected naturally or experimentally with *M. bovis* as described in sample collection (i) and (ii). The samples were tested with the rMbovP0145 iELISA, and the diagnostic sensitivity and specificity were determined based on ROC analysis using the online EpiTools (https://epitools.ausvet.com.au/roccurves?page=ROC_curves). The analytical specificity of rMbovP0145-iELISA was determined by testing the antisera against common pathogens of cattle, including *Pasteurella multocida, Brucella abortis, Salmonella, E. coli, Mycobacterium avium* sp. *paratuberculosis*, foot and mouth disease virus, infectious bovine rhinotracheitis virus, and rotavirus purchased from the China Institute of Veterinary Drug Control.

### Data Availability

The mass spectrometry proteomics data derived from this study have been deposited with the ProteomeXchange Consortium *via* the PRIDE partner repository with the data set identifier PXD017700.

### Statistical Analysis

Data were compared using one-way analysis of variance (ANOVA) followed by Tukey's multiple-comparison test. Statistical analyses were performed using GraphPad Prism 8 software (GraphPad Software, USA) with 5% significance level.

## Results

### Genome-Wide Prediction of *M. bovis* Secretomes With Bioinformatics Tools

**Figure 1:**
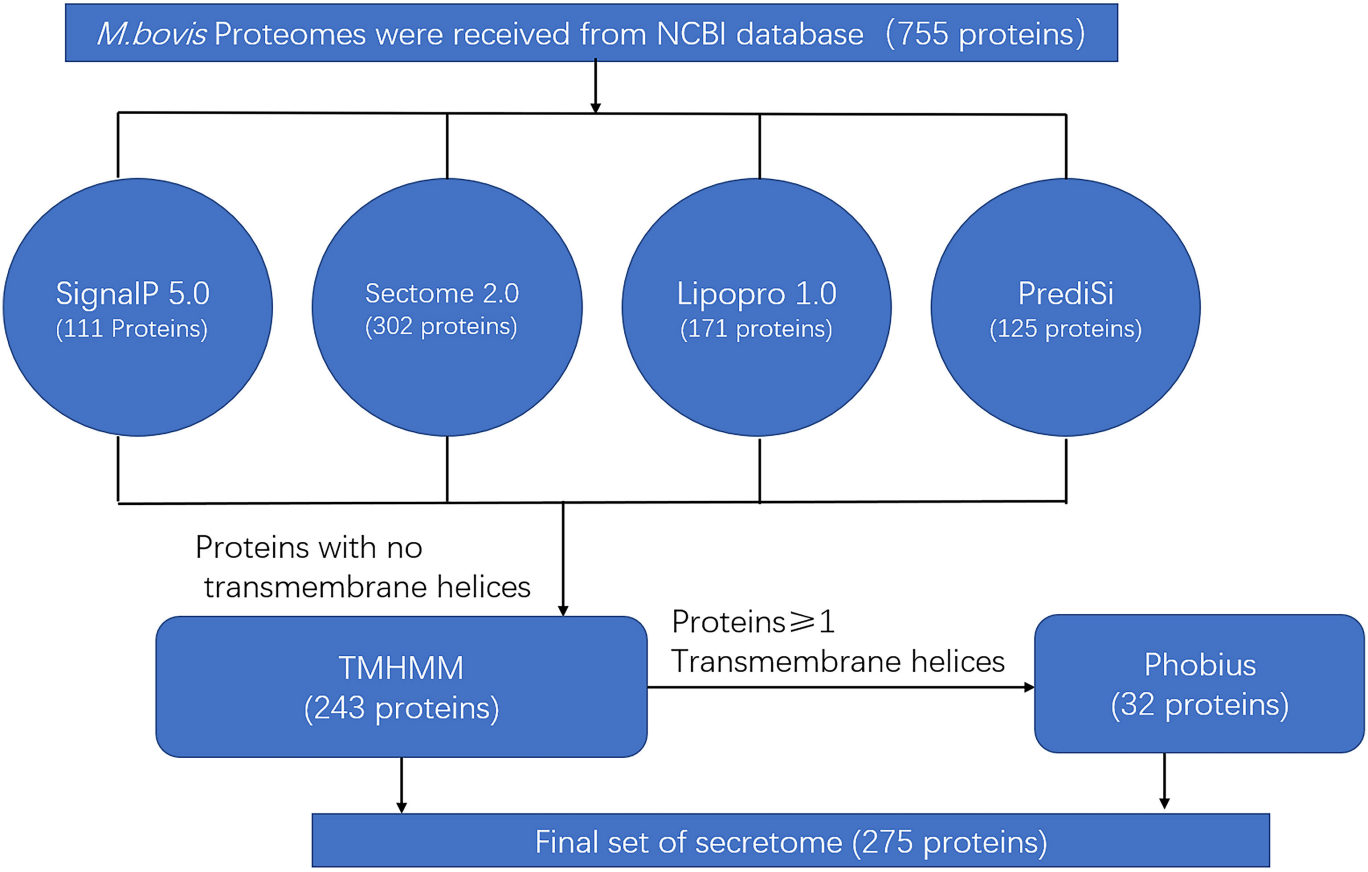
Bioinformatics pipeline to identify the secretome of *Mycoplasma bovis* genome.

The predicted secreted proteins were summarized and listed at critical points during the screening process. The NCBI database was first used to retrieve 755 proteins encoded by the genome of *M. bovis* HB0801 (P1). The four algorithms (SignalP, SecretomeP 2.0, LipoP 1.0, and PrediSi) were then merged to yield a set of 418 commonly secreted proteins. The TMHMM 2.0 algorithm was then applied to remove 175 proteins with transmembrane regions. The remaining 243 proteins were considered to represent the secretome candidates for the *M. bovis* HB0801genome. The proteins containing transmembrane motifs were also re-analyzed with Phobius to identify proteins with an α-helical conformation in the amino acid section that was previously mistakenly classified as a transmembrane region; this step added 32 more proteins with predictive signal peptides to the list of secreted proteins. The predicted secretome of P1 strain consisted of 275 proteins, representing 36.4% of the total 755 proteins of the P1 strain (Figure 1 and Supplementary Table 1).

### Label-Free Quantitative Proteomics Analysis of *Mycoplasma bovis* P1 and P150 Strains

**Figure 2:**
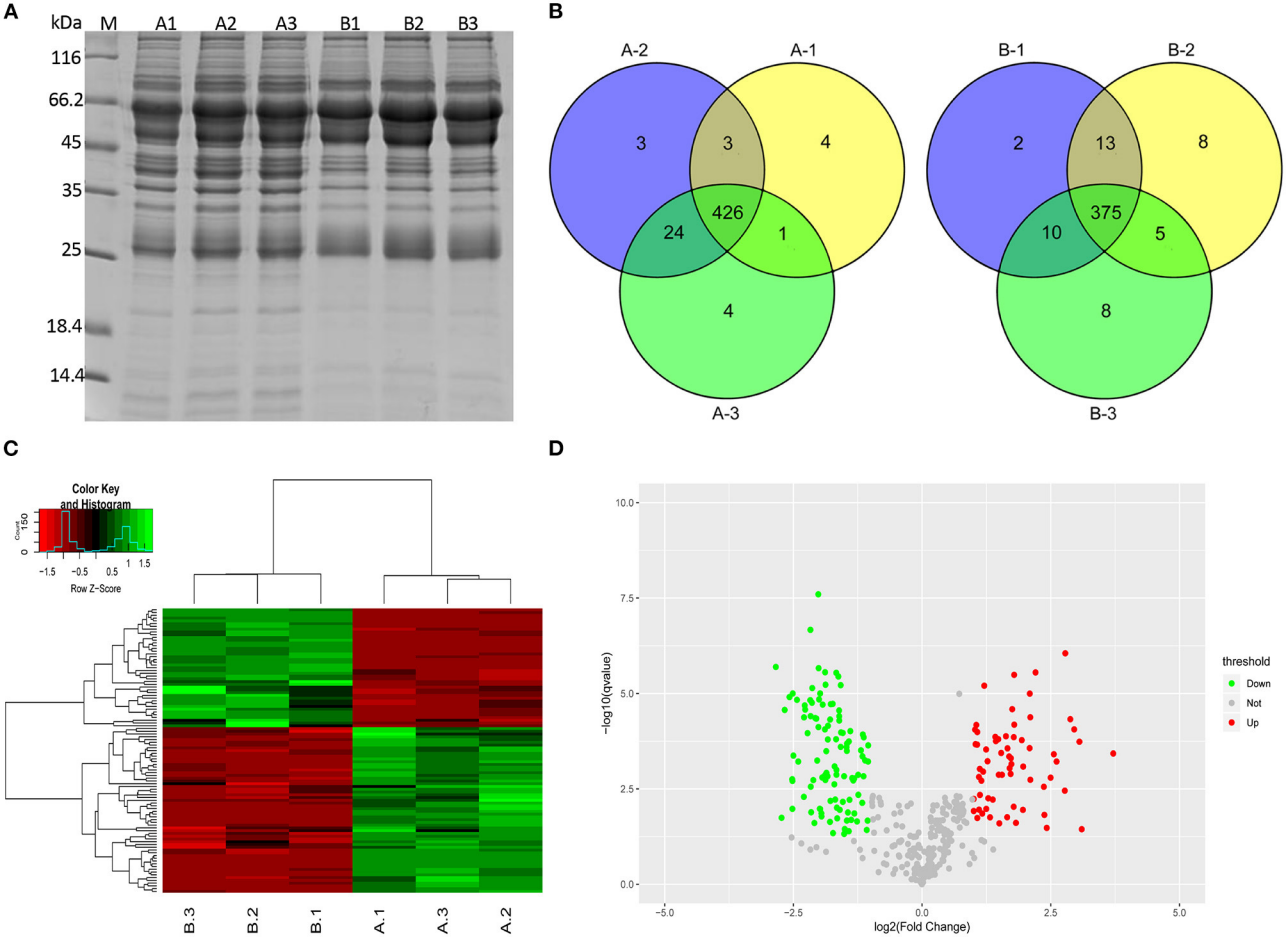
Secreted proteins were identified for *Mycoplasma bovis* P1 (virulent) and P150 (attenuated) strain by label-free quantitative proteomics analysis. **(A)** The extracted secretomes were checked with SDS-PAGE. Lanes A1, A2, and A3 and B1, B2, and B3 represent triplicate samples from *M. bovis* P1 (A1-3) and P150 (B1-3), respectively. M represents the reference protein with the molecular weight labeled on the left. **(B)** Venn diagrams of the secreted proteins from the P1 and P150 strain in triplicate. **(C)** Hierarchical cluster analysis was conducted for the differentially expressed proteins. **(D)** Distribution of differentially regulated proteins in the P1 and P150 strain. Scatter plots of log2-fold change on the x-axis against the -log *P*-value on the y-axis for significantly quantified proteins. Increased expression is labeled in red, while decreased expression is labeled in green.

An electrophoretic profile of the proteomics secreted by the two *M. bovis* strains was obtained by cultivating the strains to log phase for 18 h at 37°C in PPLO medium and then collecting the cells by centrifugation at 15,000 *g* for 20 min at 4°C. The pellet was washed with PBS, resuspended in sterile PBS, and kept for 2 h at 37°C to allow the cells to secrete proteins. During this process, the measurements of CFU by plate counting assays confirmed a stable cell viability during the PBS incubation (see Supplementary Figure 1). The culture supernatants containing the potentially secreted proteomics were then harvested from the *M. bovis* P1 and P150 strains. Images of 12% SDS-PAGE gels confirmed the good quality of the extracted protein samples, with no apparent impurities and a clear difference in protein abundance compared to *M. bovis* pellets (Figure 2A and Supplementary Figure 2). A label-free quantitative proteomic technique was used to compare the secretomes of the P1 and P150 strains. Materials <5 kDa in size were filtered out, and the filtered samples were concentrated by ultrafiltration. Triplicate protein samples from the P1 (A1, A2, and A3) and P150 (B1, B2, and B3) strains were subjected to label-free quantitative proteomics analysis; the viable *M. bovis* cells in the final preparation were confirmed as less than five viable cells/10^8^ CFU. In total, the coding sequences of 477 proteins (477/755; 63.1%) in *M. bovis* were identified from 46,027 spectra of the whole output 309,933 spectra with protein FDR ≤ 0.01 (Supplementary Table 2). This included 426 proteins from P1 strain and 375 from P150 strain that were identified in at least two out of three biological replicates (Figure 2B).

The identified proteins were hierarchically clustered (Figure 2C). A two-sample *t*-test was employed to define the proteins that were differentially regulated between P1 and P150 strains. We quantified 66 increased and 48 decreased proteins' abundance in the P1 compared to the P150. In addition, 46 proteins were exclusively identified in the P1 strain, whereas five proteins were found only in the P150 strain. All the proteins with differential abundances are listed in Supplementary Table 3. The distribution of proteins with differential abundance in the two groups is shown in Figure 2D.

### Identification of Common Proteins Between the Predicted and Extracted Secretomes

**Figure 3:**
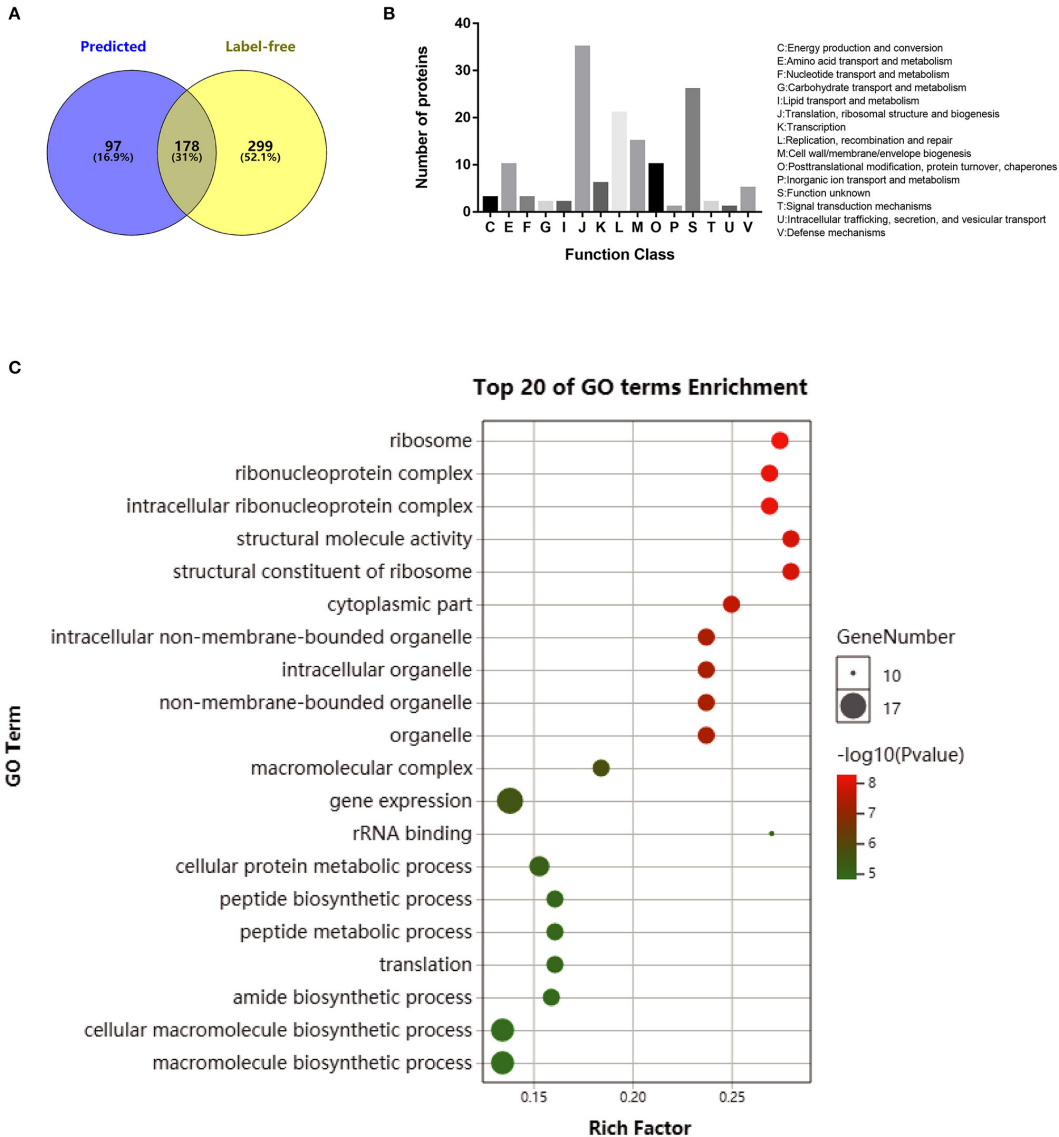
Functional analysis of overlapped secreted proteins obtained by a computation of computational prediction and label-free proteomics assays. **(A)** Venn diagrams showing the proteins obtained by genome prediction and/or identified with label-free proteomic assay for both *Mycoplasma bovis* P1 (virulent) and P150 (attenuated) strains. **(B)** Protein distribution in functional categories in the cluster of orthologous groups classification. **(C)** Gene ontology (GO) enrichment pathway analysis of the secreted proteins in *M. bovis*. Only the top 20 GO enrichments are presented. The circle size is positively associated with the number of enriched genes, and the colors depict the *p*-values.

The secreted proteins were characterized with high accuracy by identifying the probable secreted proteins based on both approaches of label-free analysis and genomic prediction. In total, 178 overlapped proteins were defined as the final secreted proteins using the principle of the greatest common divisor (Figure 3A), and these are listed in Supplementary Table 4. These proteins were further classified according to the predicted cluster of orthologous group (COG) functional terms, and 79% (141/178) of them were assigned into specific functional COG families comprising 15 functional categories. The top six categories, in decreasing order, were J (translation, ribosomal structure, and biogenesis; 35 proteins), S (function unknown; 25 proteins), L (replication, recombination, and repair; 21 proteins), M (cell wall/membrane/envelope biogenesis; 13 proteins), E (amino acid transport and metabolism; 10 proteins), and O (posttranslational modification, turnover, and chaperon; 10 proteins) (Figure 3B). GO enrichment analysis was performed for the categories of molecular function, cellular components, and biological process for the 178 secreted proteins (Supplementary Table 5), and the top 20 enriched GO terms were further analyzed based on the functions (Figure 3C). The biological process ontology indicated enrichment in cellular protein metabolic processes and translation and peptide biosynthetic processes. The molecular function ontology indicated high enrichment in the structural constituents of ribosomes, structural molecule activity, and rRNA binding. The cellular component ontology indicated high enrichment in the ribosome, ribonucleoprotein complex, and intracellular ribonucleoprotein complex.

### Comparative Functional Analyses According to Secretion Pathways and Subcellular Locations

**Figure 4:**
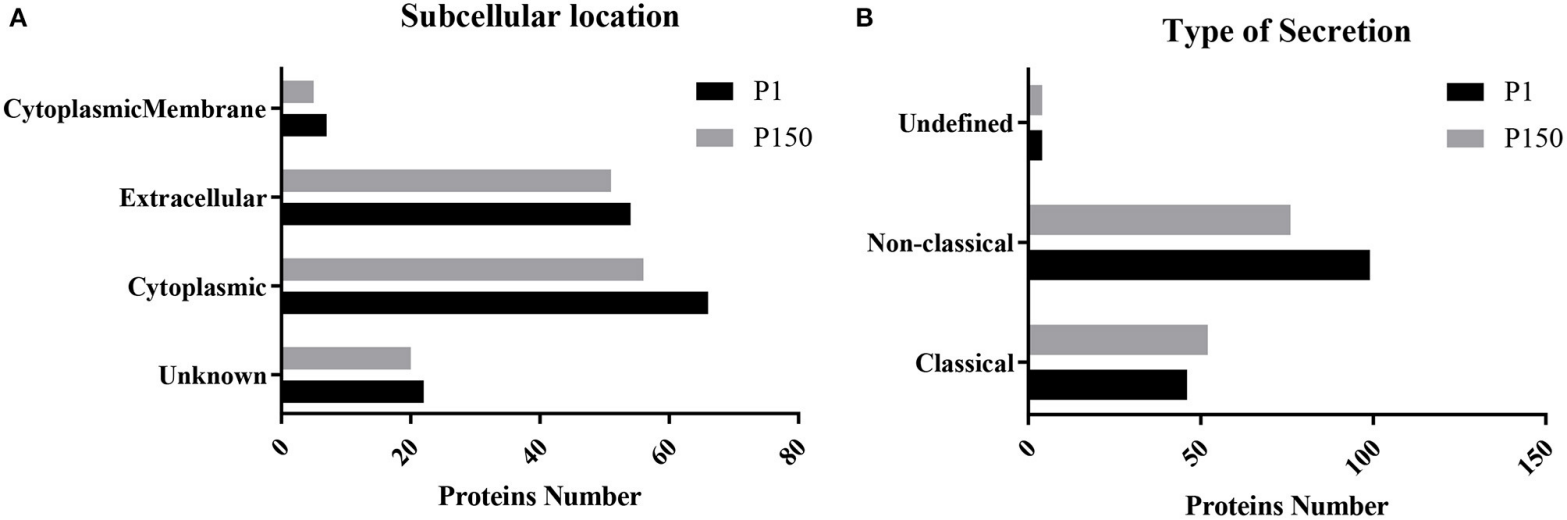
Predicted secretion pathways and subcellular locations of *Mycoplasma bovis* proteins identified in the P1 (virulent) and P150 (attenuated) strains. **(A)** Type of secretion pathway. **(B)** Predictions of subcellular location.

The qualitative and quantitative proteomic analyses of the two strains further excluded 16 proteins with one or two null data in three repetitions of the 178 potential secreted proteins. Among the remaining 162 proteins, 119 proteins were shared between P1 and P150 strains, while 30 proteins were exclusively identified in P1 and 13 in P150. Therefore, P1 was associated with 149 secreted proteins, while P150 was associated with 132 proteins. These proteins were further classified according to the types of secretion and locations. Among the 149 proteins of the P1 strain, 46 proteins were predicted as classical secreted proteins, while 99 proteins were predicted as non-classical secreted proteins, and four proteins were assigned an undefined type of secretion (Figure 4A). The subcellular locations indicated seven proteins predicted as cytoplasmic membrane proteins, 54 as extracellular proteins, 66 as cytoplasmic proteins, and 22 as unknown (Figure 4B). Among the 132 proteins of P150 strain, 52 proteins were predicted to be classical secreted proteins, while 76 were classified as non-classical secreted proteins, and four were undefined secreted types (Figure 4A). The predicted subcellular locations of the P150 proteins were five as cytoplasmic membrane proteins, 51 as extracellular proteins, 56 as cytoplasmic proteins, and 20 as unknown type (Figure 4B). In other words, the number of putative secreted proteins was close in both the P1 (149 proteins) and P150 (132 proteins) strains, and the predicted subcellular distributions and secretion types were also similar for the secreted proteins in both strains.

### Proteins With Different Abundance Between *M. bovis* P1 and P150 Strains

A quantitative analysis of the P1 and P150 proteins was performed with all the identified proteins based on the label-free proteomic data and further classified 79 proteins with differential abundance into four clusters. Among them, 17 had an increased abundance in the P1 strain compared to the P150 strain, while 42 had a reduced abundance. In addition, 17 proteins were exclusively detected in P1, while three were exclusively detected in P150 (Table 1).

**Table 1:** Seventy-nine differentially secreted proteins between P1 and P150 strains.

**Protein IDs**	**Protein description**	**Conserved domains**	**MW kDa**	**Folds in abundance P1/P150**	***T* test *p* value**	**Log2 fold change (P1/P150)**	**VirulentPred results**	**Blast *E*-values**
**Proteins more abundant in P1 (17)**								
Mbov_0145	Hypothetical protein	DUF285	55.3	53.8	0.0008	5.7	/	/
Mbov_0725	Cof-type HAD-IIB family hydrolase	Hydrolase_3	32.9	43.0	0.0000	5.4	/	/
Mbov_0618	50S ribosomal protein L24	rplX	12.1	8.6	0.0361	3.1	/	/
Mbov_0793	Variable surface lipoprotein	PTZ00449	23.6	6.8	0.0035	2.8	/	/
Mbov_0637	50S ribosomal protein L27	rpmA	10.1	5.2	0.0028	2.4	/	/
Mbov_0598	30S ribosomal protein S11	PRK05309	14.3	4.3	0.0018	2.1	/	/
Mbov_0294	30S ribosomal protein S2	rpsB	35.5	4.3	0.0000	2.1	/	/
Mbov_0076	Phenylalanine–tRNA ligase subunit beta	pheT	82.4	4.3	0.0000	2.1	/	/
Mbov_0411	Excinuclease ABC subunit UvrA	uvrA	105.5	3.9	0.0008	2.0	Virulent	3.00E-47
Mbov_0107	ISMbov1 family transposase	Tra8 super family	48.6	3.5	0.0001	1.8	/	/
Mbov_0627	50S ribosomal protein L23	rplW	16.7	3.3	0.0007	1.7	/	/
Mbov_0266	tRNA pseudouridine (55) synthase TruB	truB super family	32.3	2.8	0.0013	1.5	/	/
Mbov_0462	ISMbov3 family transposase	/	27.7	2.4	0.0056	1.3	/	/
Mbov_0333	Ribonuclease III	RNaseIII super family	25.2	2.2	0.0140	1.2	/	/
Mbov_0089	50S ribosomal protein L1	rplA	25.0	2.2	0.0015	1.1	/	/
Mbov_0820	tRNA-binding protein	tRNA_bind_bactPheRS	22.5	2.1	0.0183	1.1	/	/
Mbov_0839	Hypothetical protein	LacI/PurR family	34.0	2.8	0.0254	1.5	Virulent	4.00E-79
**Proteins more abundant in P150 (42)**								
Mbov_0458	ISMbov3 family transposase	SdrC	31.9	0.1	0.0000	−2.8	Virulent	4.00E-68
Mbov_0654	Putative lipoprotein	Efa1_rel_toxin; PRK08581	28.4	0.2	0.0000	−2.7	Virulent	3.00E-44
Mbov_0174	BMP family ABC transporter substrate-binding protein	Periplasmic binding protein type1	74.0	0.2	0.0000	−2.6	/	/
Mbov_0461	IS1634-like element ISMbov3 family transposase	/	21.5	0.2	0.0105	−2.5	/	/
Mbov_0585	Putative lipoprotein	CCDC158	59.7	0.2	0.0000	−2.5	/	/
Mbov_0579	P80 family lipoprotein	Lipoprotein_X; Lipoprotein_10	81.6	0.2	0.0000	−2.4	/	/
Mbov_0473	Variable surface lipoprotein	PRK08581	23.7	0.2	0.0001	−2.0	/	/
Mbov_0515	Variable surface lipoprotein	DUF31; SMC_prok_A	97.6	0.2	0.0000	−2.0	/	/
Mbov_0156	Variable surface lipoprotein	/	37.1	0.3	0.0105	−2.0	/	/
Mbov_0536	Putative lipoprotein	aro_clust_Mycop	45.1	0.3	0.0107	−2.0	/	/
Mbov_0570	Putative lipoprotein	PRK06148	85.5	0.3	0.0167	−2.0	/	/
Mbov_0580	Thermonuclease family protein	SNc	44.2	0.3	0.0016	−1.9	Virulent	2.00E-35
Mbov_0393	Putative membrane lipoprotein (ICEB-1 encoded)	/	59.3	0.3	0.0014	−1.9	/	/
Mbov_0838	IS1634-like element ISMbov3 family transposase	DUF285	50.3	0.3	0.0000	−1.9	/	/
Mbov_0419	Hypothetical protein	DUF2732	116.8	0.3	0.0015	−1.8	/	/
Mbov_0296	Peptidase S41	Peptidase_S41	71.0	0.3	0.0232	−1.8	/	/
Mbov_0696	Hypothetical protein	SMC_prok_A	24.2	0.3	0.0000	−1.8	Virulent	2.00E-48
Mbov_0115	ATP-binding cassette domain-containing protein	AppF; P-loop_NTPase; COG0610	94.0	0.3	0.0456	−1.7	Virulent	2.00E-80
Mbov_0517	Putative immunoglobulin-blocking virulence protein	predic_Ig_block	84.0	0.3	0.0000	−1.7	/	/
Mbov_0537	Putative lipoprotein	aro_clust_Mycop	34.1	0.3	0.0000	−1.6	/	/
Mbov_0449	Variable surface lipoprotein	P30	26.4	0.3	0.0003	−1.6	/	/
Mbov_0468	Putative lipoprotein	DUF31	65.7	0.3	0.0001	−1.6	/	/
Mbov_0516	Putative immunoglobulin-blocking virulence protein	predic_Ig_block	83.8	0.4	0.0358	−1.5	/	/
Mbov_0471	Peptidase S41	Peptidase_S41	75.1	0.4	0.0003	−1.5	/	/
Mbov_0581	ATP-binding cassette domain-containing protein	MalK;GsiA	80.2	0.4	0.0214	−1.5	Virulent	4.00E-89
Mbov_0111	Putative lipoprotein	/	110.1	0.4	0.0003	−1.5	/	/
Mbov_0548	Putative lipoprotein	/	25.7	0.4	0.0002	−1.4	/	/
Mbov_0274	Putative lipoprotein	Periplasmic_Binding_Protein_Type_2	66.5	0.4	0.0061	−1.4	/	/
Mbov_0765	MULTISPECIES: hypothetical protein	/	8.1	0.4	0.0017	−1.4	/	/
Mbov_0658	Peptidase S41	Peptidase_S41; Trypan_PARP	73.1	0.4	0.0017	−1.3	/	/
Mbov_0656	Putative lipoprotein	valS	31.3	0.4	0.0139	−1.3	Virulent	5.00E-39
Mbov_0798	Variable surface lipoprotein	/	30.4	0.4	0.0226	−1.3	/	/
Mbov_0674	Endonuclease/exonuclease/phosphatase family protein	MnuA_DNase1-like	48.6	0.4	0.0003	−1.2	/	/
Mbov_0505	Putative lipoprotein	DUF31	90.4	0.4	0.0004	−1.2	/	/
Mbov_0693	Membrane protein	/	302.4	0.5	0.0001	−1.2	/	/
Mbov_0557	NADPH flavin oxidoreductase	FMN_reductase	27.9	0.5	0.0001	−1.1	/	/
Mbov_0519	Putative immunoglobulin-blocking virulence protein	predic_Ig_block	83.4	0.5	0.0377	−1.1	/	/
Mbov_0016	BMP family ABC transporter substrate-binding protein	Periplasmic_Binding_Protein_type1	51.2	0.2	0.0020	−2.5	/	/
Mbov_0049	Putative lipoprotein	SMC_prok_A	85.6	0.3	0.0000	−1.6	Virulent	e-105
Mbov_0518	Putative lipoprotein	DUF31; MRP-S26	96.7	0.4	0.0205	−1.4	/	/
Mbov_0326	Putative secreted acid phosphatase	HAD_like	52.7	0.5	0.0006	−1.1	/	/
Mbov_0739	Putative lipoprotein	Periplasmic_Binding_Protein_Type_2	70.0	0.5	0.0215	−1.1	/	/
**P150 unique (3)**								
Mbov_0283	Variable surface lipoprotein	CobT2	28.9	/	/	/	Virulent	5.00E-50
Mbov_0339	Variable surface lipoprotein	PRK08581	34.8	/	/	/	Virulent	3.00E-52
Mbov_0368	Hypothetical protein	SMC_prok_A	33.3	/	/	/	/	/
**P1 unique proteins (17)**								
Mbov_0044	23S rRNA [guanosine(2251)-2 '-O]-methyltransferase RlmB	SpoU	26.0	/	/	/	/	/
Mbov_0075	Uracil–DNA glycosylase	UDG-F1-like	25.3	/	/	/	Virulent	e-101
Mbov_0079	Aminotransferase class V-fold PLP-dependent enzyme	CsdA	43.4	/	/	/	/	/
Mbov_0139	50S ribosomal protein L32	rpmF	7.7	/	/	/	/	/
Mbov_0207	Hypothetical protein	/	26.6	/	/	/	/	/
Mbov_0210	Hypothetical protein	/	40.9	/	/	/	Virulent	4.00E-46
Mbov_0376	SocA family protein	DUF4065	17.4	/	/	/	/	/
Mbov_0486	Bifunctional DNA-formamidopyrimidine glycosylase/DNA-(apurinic or apyrimidinic site) lyase	PRK01103	32.4	/	/	/	/	/
Mbov_0647	DUF4011 domain-containing protein	MTES_1575; DUF4011; DEXXQc_SF1; DNA2; AAA_11	183.3	/	/	/	/	/
Mbov_0718	L-ribulose-5-phosphate 4-epimerase	araD	27.3	/	/	/	/	/
Mbov_0732	DUF285 domain-containing protein	DUF285	38.0	/	/	/	/	/
Mbov_0750	ATP-binding protein	P-loop_NTPase	34.7	/	/	/	Virulent	e-100
Mbov_0795	Variable surface lipoprotein	PTZ00449	22.0	/	/	/	Virulent	2.00E-3
Mbov_0848	AAA family ATPase	recD_rel	87.2	/	/	/	Virulent	e-133
Mbov_0034	ABC transporter ATP-binding protein	DppD	61.4	/	/	/	Virulent	e-131
Mbov_0108	50S ribosomal protein L28	rpmB	7.4	/	/	/	/	/
Mbov_0116	Hypothetical protein	/	37.4	/	/	/	/	/

The putative novel virulence factors were predicted by further analyzing these differential proteins using PSI-BLAST in VirulentPred. This revealed 18 proteins that were classified as virulent, including two more abundant (MbovP0411 and MbovP839) and eight less abundant (MbovP0049, MbovP0115, MbovP0458, MbovP0580, MbovP0581, MbovP0654, MbovP0656, and MbovP0696) in P1 than in P150. Another eight proteins were also exclusively detected in only one strain, including six exclusively in P1: a uracil-DNA glycosylase (MbovP0075), two ATP-binding proteins (MbovP0034 and MbovP0750), an AAA family ATPase (MbovP0848), and two hypothetical proteins (MbovP0210 and MbovP0795). The other two were variable surface proteins (MbovP0283 and MbovP0339), which are major lipoprotein antigens and were found exclusively in the P150 strain. The proteins with observed qualitative differences in abundance are presented in Table 1. Although some other proteins did not give hits in the VirulentPred database, their significant differential expression suggests their possible importance. Among them, the abundance of a hypothetical protein, MbovP0145, and a hydrolase of Cof-type HAD-IIB family, MbovP0725, was 53.8 and 43.0 times higher, respectively, in P1 than in P150 (Table 1).

### Fourty-Nine Secreted Proteins Were Found in the Core Genome Content

The core genes in mycoplasma genomes were highly conserved during evolution and are responsible for the basic biological characteristics; therefore, the core genes that possibly encoded secreted proteins were further analyzed. Our previous pan-genome prediction of 13 different species of *Mycoplasma* indicated that 224 core genes are present in all of them (21). Of those genes, 21.8% (49/224) were present in our list of 178 secreted proteins and might be involved in essential cellular functions, as shown by 61% (30/49) of the proteins belonging to J category (translation, ribosomal structure, and biogenesis), while the proportions of L, O, and K categories were 12% (6/49), 8% (4/49), and 6% (3/49), respectively (Table 2). Of the 49 core genes, 16 were differentially abundant (Table 2). Interestingly, six proteins were only existent in P1, and nine proteins were more abundant in P1 than in P150. The expression of these core genes might be related to the pathogenesis of the *M. bovis* strain.

**Table 2:** The core secreted proteins encoded by 49 core genes of 13 mycoplasmas.

**Gene ID**	**Protein description**	**COG**	**Different expression**
Mbov_0044	23S rRNA (guanosine(2251)-2\\'-O)-methyltransferase RlmB	J	P1 unique
Mbov_0048	Transcription termination/antitermination protein NusG	K	/
Mbov_0058	TatD family hydrolase	L	/
Mbov_0075	Uracil–DNA glycosylase	L	P1 unique
Mbov_0076	Phenylalanine–tRNA ligase subunit beta	J	Increased abundance
Mbov_0079	Aminotransferase class V-fold PLP-dependent enzyme	E	P1 unique
Mbov_0080	Iron–sulfur cluster assembly scaffold protein	C	/
Mbov_0088	50S ribosomal protein L11	J	/
Mbov_0089	50S ribosomal protein L1	J	Increased abundance
Mbov_0108	MULTISPECIES: 50S ribosomal protein L28	J	P1 unique
Mbov_0115	ATP-binding cassette domain-containing protein	P	Decreased abundance
Mbov_0139	50S ribosomal protein L32	J	P1 unique
Mbov_0214	30S ribosomal protein S20	J	/
Mbov_0220	MULTISPECIES: 50S ribosomal protein L33	J	/
Mbov_0234	Ribosome small subunit-dependent GTPase A	S	/
Mbov_0269	30S ribosomal protein S15	J	/
Mbov_0294	30S ribosomal protein S2	J	Increased abundance
Mbov_0302	RNA polymerase sigma factor	K	/
Mbov_0303	DNA primase	L	/
Mbov_0316	30S ribosomal protein S18	J	/
Mbov_0333	Ribonuclease III	J	Increased abundance
Mbov_0370	tRNA 2-thiouridine (34) synthase MnmA	J	/
Mbov_0411	Excinuclease ABC subunit UvrA	L	Increased abundance
Mbov_0432	Leucine–tRNA ligase	J	/
Mbov_0433	Endopeptidase La	O	/
Mbov_0452	Tryptophan–tRNA ligase	J	/
Mbov_0479	Hypothetical protein	O	/
Mbov_0486	Bifunctional DNA-formamidopyrimidine glycosylase/DNA lyase	L	P1 unique
Mbov_0520	NAD-dependent DNA ligase LigA	L	/
Mbov_0529	30S ribosomal protein S9	J	/
Mbov_0542	FMN-dependent NADH-azoreductase	I	/
Mbov_0551	MULTISPECIES: 50S ribosomal protein L20	J	/
Mbov_0576	Thioredoxin	/	/
Mbov_0598	MULTISPECIES: 30S ribosomal protein S11	J	Increased abundance
Mbov_0612	30S ribosomal protein S5	J	/
Mbov_0618	MULTISPECIES: 50S ribosomal protein L24	J	Increased abundance
Mbov_0621	50S ribosomal protein L29	J	/
Mbov_0625	30S ribosomal protein S19	J	/
Mbov_0627	50S ribosomal protein L23	J	Increased abundance
Mbov_0628	50S ribosomal protein L4	J	/
Mbov_0630	30S ribosomal protein S10	J	/
Mbov_0637	50S ribosomal protein L27	J	Increased abundance
Mbov_0641	Nucleotide exchange factor GrpE	O	/
Mbov_0643	50S ribosomal protein L31	J	/
Mbov_0678	MULTISPECIES: 30S ribosomal protein S12	J	/
Mbov_0766	DUF402 domain-containing protein	J	/
Mbov_0786	Transcription termination/antitermination protein NusA	K	/
Mbov_0807	Asp-tRNA(Asn)/Glu-tRNA(Gln) amidotransferase subunit GatA	J	/
Mbov_0817	DnaJ domain-containing protein	O	/

*COG, cluster of orthologous groups (database functional classes); C, energy production and conversion; I, lipid transport and metabolism; J, translation, ribosomal structure, and biogenesis; K, transcription; L, replication, recombination, and repair; O, post-translational modification, protein turnover, and chaperones; P, inorganic ion transport and metabolism; S, function unknown; /, no difference*.

### Validation of Three Proteins With Differential Abundance Between P1 and P150 Strains

**Figure 5:**
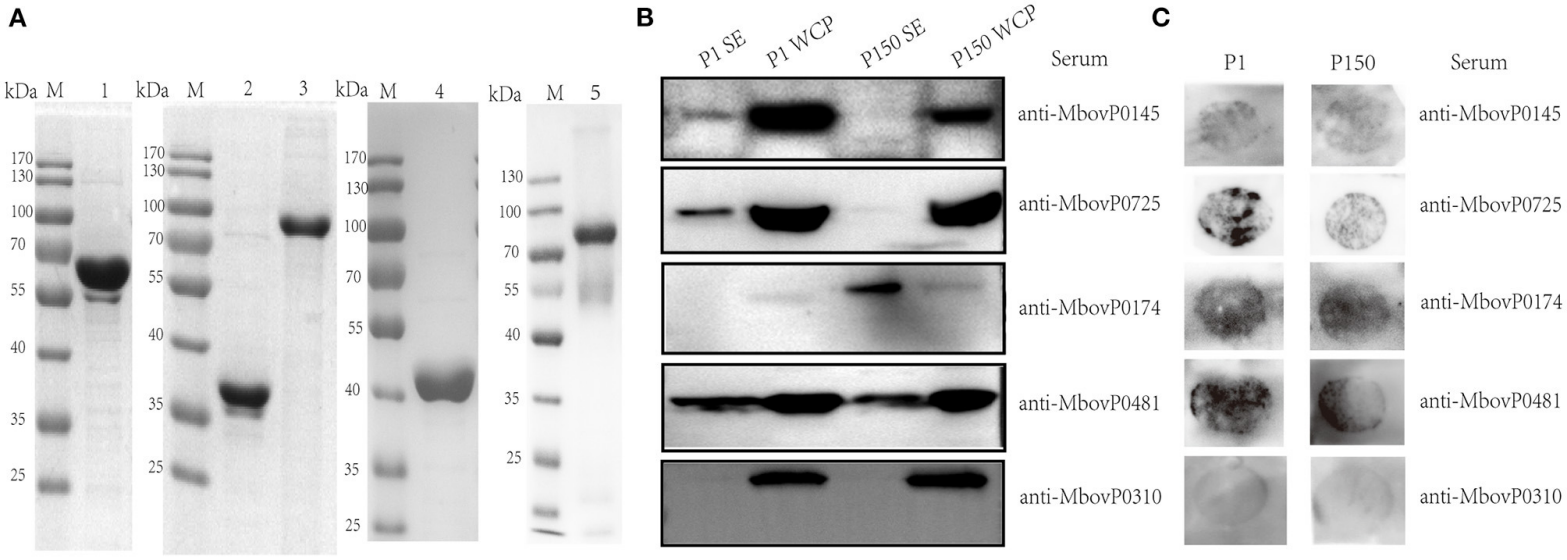
Verification of the secreted nature of five *Mycoplasma bovis* proteins. **(A)** Five proteins were expressed, purified, and confirmed by 12% SDS-PAGE. Lane 1: rMbovP0145, lane 2: rMbovP0725, lane 3: rMbovP0174, lane 4: rMbovP0481, and lane 5: rMbovP0310. M represents the protein marker. **(B)** Western blot verification of the secreted nature of the selected proteins in the *M. bovis* P1 (virulent) and P150 (attenuated) strains. SE represents the secretomes, while WCP represents the whole cell proteins extracted from the *M. bovis* P1 and P150 strains. **(C)** Colony immunoblotting assay to detect the presence of the four selected proteins on the mycoplasma surface. The colonies were incubated with antisera to each protein.

Based on the log_2_fold changes, we selected three proteins—MbovP0145, MbovP0725, and MbovP0174—with differential abundance between P1 and P150 strains to confirm their secretion and differential expression. We used MbovP0481 as the positive control and MbovP0310 as the negative control. These genes were modified according to *E. coli* codon bias, cloned, expressed in *E. coli*, and purified based on the His-tags. The molecular masses with the His-tags for MbovP0145, MbovP0725, MbovP0174, MbovP0481, and MbovP0310 were consistent with the expected values (Figure 5A). The mouse antiserum for each recombinant protein was developed with titers of over 4 × 10^5^ (Supplementary Figure 3). As shown in Figure 5B, the selected proteins were differentially detected between the supernatant fraction and the WCP from the cellular pellets, and their alterations in abundance between P1 and P150 were consistent with the results of the quantitative proteomic analysis. Since the colony immunoblotting assay could detect the expression of the surface and secreted proteins on *M. bovis*, it demonstrated that MbovP0145, MbovP0725, MbovP0174, and MbovP0481, rather than MbovP0310, are either secreted or surface proteins (Figure 5C), which is in agreement with the data in Figure 5B.

### Antigenicity of the Secreted Proteins With Different Abundance

**Figure 6:**
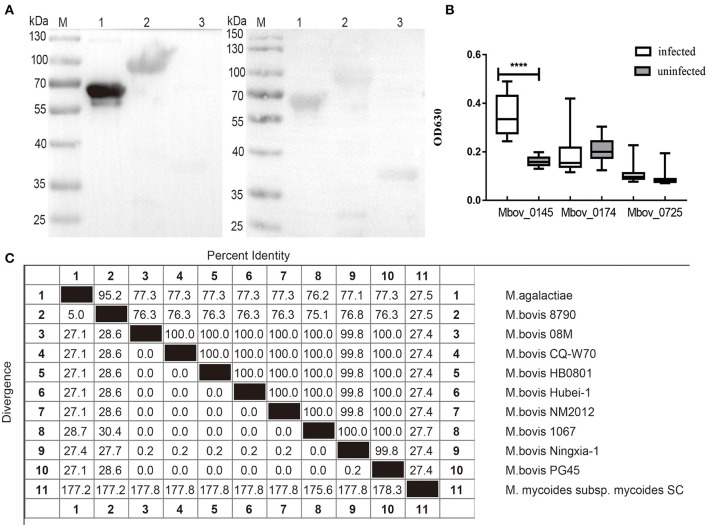
Evaluation of the reaction of rMbovP0145, rMbovP0174, and rMbovP0725 with the pooled cattle sera positive and negative for *Mycoplasma bovis* infection. **(A)** Western blotting detected the reaction of the recombinant proteins with the pooled sera (diluted 1:100) from 15 *M. bovis* infected and 15 uninfected calves. M, protein marker; lane 1, MbovP0145; lane 2, MbovP0174; lane 3, MbovP0725. **(B)** iELISA tests of the reaction of the recombinant proteins with individual serum samples (diluted 1:100) from 15 *M. bovis* infected and 15 uninfected calves. The horizontal solid lines for each group indicate the median values; ^****^p < 0.0001. **(C)** Amino acid sequence identity (%) of MbovP0145 sequences among *Mycoplasma* species in this study. Their respective Genebank accession numbers are as follows: *Mycoplasma agalactiae* (WP_041308700.1), *M. bovis* 8790 (TKA58782.1), *M. bovis* 08M (WP_013954588.1), *M. bovis* CQ-W70 (AIA33730.1), *M. bovis* Hubei-1(AEI89852.1), *M. bovis* NM2012 (WP_013954588.1), *M. bovis* 1067 (TKA60039.1), *M. bovis* Ningxia-1 (WP_141790766.1), *M. bovis* PG45 (WP_080551560.1), and *M. mycoides* subsp. *mycoides SC* (WP_011166602.1).

Western blotting assay was used to detect the reaction of the three confirmed differentially secreted proteins rMbovP0145, rMbovP0174, and rMbovP0725 with the pooled cattle sera positive or negative for *M. bovis* infection. The rMbovP0145 showed a stronger reaction to the positive serum than to the negative serum. The other two proteins showed no difference in their reactions between the positive and negative sera (Figure 6A). Furthermore, the iELISA reactions, run by coating the three proteins on wells of ELISA plates, also confirmed their immunogenicity. The rMbovP0145 generated the strongest reaction to the positive serum but had a very weak reaction to the negative serum (*p* < 0.001) (Figure 6B).

We also performed searches for orthologs of MbovP0145 from different *Mycoplasma* species and other mycoplasmas. The results demonstrated that MbovP0145 is highly conserved in most *M. bovis* strains, with the exception of *M. bovis* 8790 (76.3% of identity) (GenBank accession no. NZ_LAUS01000004) isolated from a caprine in Ethiopia, while sequences from *Mycoplasma agalactiae* (77.3% of identity) and *M. mycoides* subsp. *mycoides SC* (27.4% of sequence identity) present lower amino acid identities (Figure 6C). Despite the low conservation of the complete sequences, the DUF285 domain from all strains was conserved, indicating that it might display similar functions in various mycoplasma species.

The conservation of MbovP0145 in different *M. bovis* strains was confirmed by examining the whole cell protein of 11 *M. bovis* clinical isolates, as well as P1 and P150 strains, by western blotting assay. This protein was stably and strongly expressed in all clinical strains but only weakly expressed in P150 (Supplementary Figure 4).

### Preliminary Evaluation of A rMbovP0145-Based iELISA

**Figure 7:**
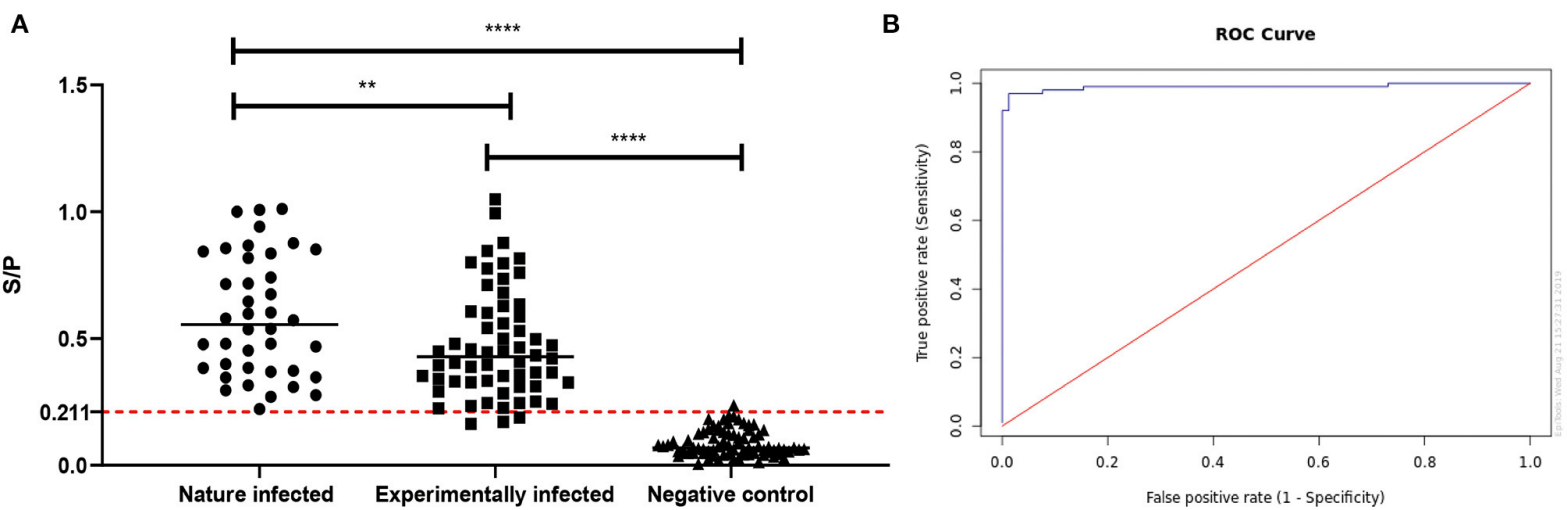
Evaluation of the rMbovP0145-based iELISA for the detection of an IgG response to *Mycoplasma bovis* infection. **(A)** Comparison of serum antibody detection with rMbovP0145-iELISA from naturally infected, experimentally infected, and uninfected cattle groups. The cutoff for the sample-to-positive (S/P) value was determined as 0.211 when the overall diagnostic sensitivity and specificity were 97.0 and 98.7%, respectively. The bar indicates the mean of each group, and the dashed line indicates the cutoff point. The diagnostic sensitivity of 98.7% (95% CI: 93.1–99.8%) and specificity of 97.5% (95% CI: 87.1–99.6%) for the natural infection are significantly higher than the sensitivity of 89.7% (95% CI: 81–94.7%) and specificity of 90% (95% CI: 79.9–95.3%) for the experimental infection (^**^p < 0.01). Both two groups were significantly higher than the negative controls, respectively (^****^p < 0.0001). **(B)** Receiver operating characteristic analysis of the rMbovP0145-iELISA.

The rMbovP0145-based iELISA was developed by optimizing critical parameters, including the concentration of the coated antigen at 100 ng/well and the serum (positive and negative) dilution at 1:100. The potential application of this iELISA as a diagnostic biomarker was then evaluated. Briefly, a total of 100 positive serum samples, including 40 from naturally infected and 60 from experimentally infected cattle, as well as 78 negative serum samples from uninfected cattle were tested, and the S/P values were calculated. The ROC analysis confirmed the S/P cutoff value of 0.211 for detection of virulent *M. bovis* infection when the overall diagnostic sensitivity was 97.0% (95% CI: 91.5–99.0%), the diagnostic specificity was 98.7% (95% CI: 93.1–99.8%), and the area under the curve value was 0.99 (95% CI: 0.975–1.00) (Figures 7A,B). A comparison of the validity of this technique for the detection of natural and experimental infection revealed a diagnostic sensitivity of 98.7% (95% CI: 93.1–99.8%) and specificity of 97.5% (95% CI: 87.1–99.6%) for the natural infection, and these values were significantly higher than the sensitivity of 89.7% (95% CI: 81–94.7%) and specificity of 90% (95% CI: 79.9–95.3%) for the experimental P1 infection (*p* = 0.004) (Figure 7A). Eight antisera positive against common cattle pathogens, including *Salmonella* sp., *E. coli K99, Brucella* sp., *Pasteurella multocida, Mycobacterium paratuberculosis*, infectious bovine rhinotracheitis virus, foot and mouth disease virus, and rotavirus spp., were negative in this rMbovP0145-based iELISA (Supplementary Table 6). Taken altogether, these data demonstrated that the rMbovP0145 protein could potentially be used as a target with high sensitivity and specificity for the detection of a specific antibody response induced by natural *M. bovis* and experimental P1 strain infections.

### Differential Detection of Antibody Responses Induced by Immunization

**Figure 8:**
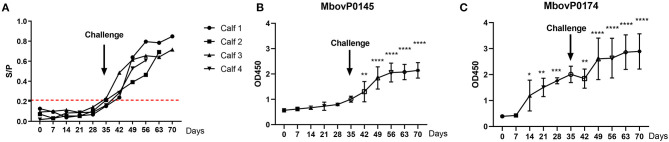
Kinetics of the serum antibody response to a post-immunization *Mycoplasma bovis* challenge. Four calves were immunized with attenuated *M. bovis* P150 for 35 days and then challenged with the wild *M. bovis* P1, followed by observation for a further 35 days. **(A)** The rMbovP0145-iELISA as the S/P value was used for the kinetic detection of *M. bovis*-positive in serum. The cutoff value is set at 0.211 as red dotted lines. **(B,C)** Comparison of the serum antibody response, expressed as OD_450_ values, induced by MbovP0145 and MbovP0174. Data represent means ± SD. Significant differences in the response *vs*. 0 days were identified using a one-way ANOVA test. ^*^p < 0.05, ^**^p < 0.01, ^***^p < 0.001, ^****^p < 0.0001.

The rMbovP0145-iELISA was also used for the kinetic detection of the antibody increase in sera from the calves after immunization and a subsequent challenge using the MbovP0174 protein that was more highly expressed in P150 as the control. The S/P values of serum antibody response showed that all serum samples collected during immunization between 0 and 35 days post-immunization (dpi) were negative for this test. However, beginning at 7 days after the challenge (equal to 42 days after immunization), the antibody titers to rMbovP0145 conversed to be positive and maintained a rising trend until 28 days after challenge (or 70 days after immunization) when the observation was terminated (Figure 8A). These findings demonstrated that this test could efficiently detect the antibody (IgG) responses induced by an infection with the virulent P1 strain but not the vaccination with the attenuated P150 strain.

The serum antibody response was also expressed by comparing the OD_450_ values induced by rMbovP0145 and rMbovP0174. The serum IgG response to rMbovP0145 did not show a significant increase during the 35 days of P150 immunization prior to the P1 virulent challenge. By contrast, the serum IgG response to MbovP0174 showed a significant increase at 14 days post-immunization (Figures 8B,C), further demonstrating that rMbovP0145 is a good diagnostic marker.

## Discussion

This study presents the first identification and comparison of the secreted proteomic profiles of the virulent *M. bovis* P1 strain and its highly passaged attenuated P150 strain using bioinformatic prediction and label-free proteomic analysis. We identified a total of 178 potentially secreted proteins as well as 79 proteins that were differentially secreted between the virulent and attenuated strains. These proteins might play critical roles as candidate virulence determinants or as protective antigens. One differently secreted protein, MbovP0145, was also used to establish an iELISA with high sensitivity and specificity for serological detection of infection with the *M. bovis* virulent strain.

### A Total of 178 Secreted Proteins Were Identified in *M. bovis* Virulent and Its Attenuated Strains

We identified a total of 477 proteins in the culture supernatants of the two strains. Then, we found that there is a large difference between the genetic prediction and secretome analysis. The non-secreted proteins are inevitably released from cell degradation or leaking due to the stimulation by PBS stress and a few *M. bovis* cells remained in the preparation. To exclude that, we extracted the greatest common divisor generated from these two approaches. Following this protocol, 178 overlapped proteins were identified, and 35 secreted proteins were previously identified with the 2-DE-based proteomic assay of *M. bovis* (27). Therefore, compared to the label-free proteomic technique, the conventional 2-DE-based proteomic assay missed many secreted proteins.

Furthermore, 178 secreted proteins were distributed into the functional categories involved in J (translation, ribosomal structure, and biogenesis), S (function unknown), L (replication, recombination and repair,), M (cell wall/membrane/envelope biogenesis), E (amino acid transport and metabolism,) and O (posttranslational modification, turnover and chaperon). The proteolytic enzymes are mostly likely to be secreted in other mycoplasma species. For example, serine protease S41 with extracellular caseinolytic activity was released into the supernatant of *M. mycoides* subsp. *capi* strain cultures (15). Interestingly, our results contained three serine proteases which were encoded by Mbov_0296, Mbov_0471, and Mbov_0658. Besides this, several lipoprotein and variable surface membrane proteins were also detected in the present study and might be released through EVs. Previous studies have reported a wide distribution of EV structures in mycoplasmas, which may transport intracellular and membrane proteins out of mycoplasma cells (28). *M. bovis* was demonstrated to release EVs under nutritional stress conditions, and the proteins of EV membranes included lipoproteins like P80 lipoprotein and some variable surface lipoproteins (24). Our secretomes contained proteins released from EVs because we used the whole supernatant of *M. bovis* culture in PBS for the extraction of the secreted proteins.

We previously identified 224 core genes belonging to *M. bovis* core genomes (21). On this basis, 49 of these secreted protein genes belonged to the core genome. The COG analysis revealed that they were mainly associated with categories J (translation, ribosomal structure, and biogenesis), K (transcription), and C (energy production and conversion) (21). Although 61% (30/49) of these proteins belong to category J involved in translation, ribosomal structure, and biogenesis, a small fraction of core secreted proteins are indeed hydrolytic enzymes which are commonly considered to be qualified as secreted proteins such as MbovP0058, MbovP0075, MbovP0076, MbovP0432, MbovP0452, and MbovP0520.

Theoretically, category J proteins are intracellular proteins which are not eligible as secreted proteins. However, some similar studies on secretomes of other mycoplasma species have also reported the presence of category J proteins in the secretome. For example, Paes et al. found that the J category (only including translocation there) of proteins comprised 7% of the *in vitro* secreted proteins in *M. hyopneumonia* and 13% of those proteins in *M. flocculare* (18). Another study showed that more than 1,000 out of the 3,619 proteins observed on the cell surface lack the transmembrane alpha-helices or transmembrane beta-barrels found in integral membrane proteins and also lack the signal peptides found in proteins secreted through the Sec pathway (29). Therefore, those intracellular proteins might be released from mycoplasma cells through EVs (24), but this possibility remains to be verified in the future.

### Seventy-Nine Proteins With Differential Abundance Between P1 and P150 Might Shed Light on the Pathogenesis of *M. bovis*

The combined findings from our qualitative and quantitative analyses identified 79 differentially secreted proteins. These differences between the P1 and P150 strains could be related to the virulence of virulent P1 or to the protective immunity of P150 strain. Of these proteins, MbovP0725 and MbovP0145 had a higher abundance in P1, as verified experimentally. MbovP0725 was annotated as a hydrolase belonging to HAD super family. In *E. coli*, a HAD phosphatase could catalyze small phosphordonors which serve as substrates for autophosphorylation of the receiver domains in the two-component signal transduction systems (30). The VirulentPred prediction identified 18 potential virulence factors that were notably up-represented and unique in P1 and include uvrA excinuclease ABC subunit (MbovP0411), two ABC transporters (MbovP0750 and MbovP0034), uracil-DNA glycosylase (MbovP0075), AAA family ATPase (MbovP0848), and two hypothetical proteins. Among them, uvrA was previously shown to be associated with DNA repair and thereby plays a possible role in bacterial survival in the hosts by eliminating mismatched or improperly integrated uracil during DNA replication to maintain genomic integrity (31). The members of the ABC transporter protein family are well-spread in both Gram-positive and Gram-negative bacteria and have important functions in the importation and secretion of many molecules across the cell membrane (32). However, ABC transporter substrate-binding proteins are usually linked to the cell membrane by a lipopeptide and therefore not qualified to be secreted proteins either. In fact, several ABC transporter proteins were found in the secretomes of *M. hyopneumoniae* (18), and the ABC transporter protein MbovP0581 from *M. bovis* was previously validated as secreted protein by this lab (27). Furthermore, MbovP581 is a highly immunogenic protein (27) and differentially expressed at a higher level in P150 than in P1, as confirmed in the present study. Besides this, the current study confirmed another ABC transporter protein, MbovP0174, to be secreted and was more abundant in P150 than in P1. As discussed above, the ABC transporter proteins might be released through EVs, but this hypothesis remains to be confirmed in the future. Overall, the differentially expressed proteins predicted in the current study might provide key clues to explain the pathogenesis of *M. bovis*.

### The Potential Application of rMbovP0145 in the Determination of *M. bovis* Infection

MbovP0145 was identified as highly secreted by P1, but not by P150, by western blotting assay and immunoreactive to *M. bovis*-positive calf sera from experimental infection with virulent P1 or natural infection. However, the IgG response after P150 immunization did not increase significantly during 35 days of observation, indicating its potential application as a DIVA antigen. The mechanism for this differentiation might mainly reflect its much higher secretion by the P1 strain than by the P150 strain. However, we cannot exclude the possibility that a phenotypic variation in P150 might partially contribute to the weak response to MbovP0145 during P1 immunization.

A BLAST search found that MbovP0145 is a homolog of the Bacteroides surface protein family (BspA), which belongs to the leucine-rich repeat (LRR) protein family and has the LRR domain and a bacterial immunoglobulin-like domain (33). In addition, a conserved “DUF285” domain with unknown function was found in MbovP0145. The gene fragment encoding the DUF285 motif was recently described as having moved among diverse bacteria by horizontal gene transfer, especially in ruminant *Mycoplasma* (sub)species (34). The BspA protein displays multiple biological activities *in vitro*, including bacterial adherence and invasion, fibronectin and fibrinogen binding, and inflammation, and it might have arisen as a consequence of lateral gene transfer from bacteria into protozoan genomes (33). For several parasites, BspA domain-containing proteins are universal and are involved in the trafficking of secreted proteins and increased adhesion (35). These functions in MbovP0145 might be associated with the high level of expression and immunogenicity during *M. bovis* P1 infection and the observed decreased after P150 immunization, implying that MbovP0145 might be a potential virulence-related factor. The rMbovP0145-based iELISA detected seroconversion at 7 days after an experimental challenge with the P1 strain. Since MbovP0145 is a conserved secreted protein among *M. bovis* strains, it could serve as a sensitive and specific biomarker for the detection of virulent *M. bovis* infection and may represent a good target for studying the pathogenesis elicited by *Mycoplasma* species.

### Decontamination of Non-secreted Proteins

The most critical issue to identify secreted proteins is the removal of contaminating proteins arising from non-secretion sources. Compared to the commonly cultured bacteria, *M. bovis* is well-known to grow slowly and to require a rich medium with a high level of proteins derived mainly from supplements like horse serum. We attempted to decrease the contamination by medium proteins by incubating *M. bovis* in protein-free PBS for a short time (2 h). Subsequent separation of the PBS supernatants from *M. bovis* cell by centrifugation and passing through 0.22-μm filters further purified the secretome fraction. We then needed to decrease the contamination of non-secreted proteins from *M. bovis* itself in response to the PBS culture stress. The preparation quality was checked by SDS-PAGE and mycoplasma plate counting assay to evaluate the degree of contamination by any remaining viable *M. bovis* cells. We set the criterion that the ratio of number of *M. bovis* cells remaining in the final preparation to the original cell number in the culture should be less than five cells per 10^8^ original cells. Compared to the data in the published paper (24), where the ratio of contaminated *M. bovis* cells in the preparation was 1,068/1.9 × 10^8^, ours is a stricter standard.

The contaminated mycoplasma proteins might also originate mainly from the degradation of the dead cells during 2 h of incubation in PBS and from protein leaking induced by PBS stimulation for 2 h. No ideal method exists yet for separating secreted from non-secreted proteins in the same system. In our previous research (27), we developed a workflow that combined this shotgun proteomic analysis of secretomes with bioinformatic prediction using several online tools, such as secretomeP and SignalP, and we identified the overlapped proteins as secreted proteins. Our verification trial demonstrated that this was a feasible and effective way to differentiate secreted proteins. Therefore, we used this approach in the present study, but we replaced the proteomic technique with a label-free proteomic technique to obtain greater numbers of secreted proteins while also exploring the nature of the differently secreted proteins between virulent wild-type P1 and its attenuated P150 strain. The use of online bioinformatic tools greatly facilitates the identification of secreted proteins from pathogenic organisms, as searches could be conducted for the signal peptide and membrane anchoring features on the sequences of hypothetical proteins encoded in their genomes of pathogenic strains (36). This procedure has been successfully applied on several other *Mycoplasma* species such as *M. hyopneumoniae* and *M. flocculare* (18). However, the nature of these proteins and their associated secretion mechanisms still require confirmation in the future.

## Data Availability Statement

The data presented in the study are deposited in the ProteomeXchange repository via PRIDE, accession number PXD017700.

## Ethics Statement

The animal study was reviewed and approved by HZAUMO-2018-027, the Committee on the Ethics of Animal Experiments at Huazhong Agricultural University. Written informed consent was obtained from the owners for the participation of their animals in this study.

## Author Contributions

HZ, YC, CH, and AG: conceptualization. HZ and GH: data curation. HZ: formal analysis and writing—original draft. AG: funding acquisition, resources, project administration, writing—review, and editing. HZ, XC, and LY: methodology. GZ, MZ, and JC: software. HC and AG: supervision. DL and YZ: validation. All authors contributed to the article and approved the submitted version.

## Conflict of Interest

The authors declare that the research was conducted in the absence of any commercial or financial relationships that could be construed as a potential conflict of interest.

## Supplementary Material

Supplementary Figure 1 Kinetic measurement of *M. bovis* growth during phosphate-buffered saline incubation.

Supplementary Figure 2 Electrophoretic images of *M. bovis* P1 and P150 culture supernatants and the whole cell proteins from the cellular pellets.

Supplementary Figure 3 The titers of mouse monospecific antisera against rMbovP0145, rMbovP0725, rMbovP0174, and rMbovP0481 (EF-Tu).

Supplementary Figure 4 Analysis of the conservation of MbovP0145 in different *Mycoplasma bovis* strains by western blotting assay. A total of 20 μg of whole cell proteins from each of the 11 *M. bovis* clinical isolates as well as P1 and P150 strains were loaded into the lanes, subjected to 12% SDS-PAGE, and transferred to a polyvinylidene difluoride membrane. The mouse antiserum was then used to probe for MbovP0145 in each strain, and the signal was developed. M, molecular mass marker; lanes 1–14, *M. bovis* strain P1, P150, NNH, EZ-8, DY, EZ-2, XM, 1834, YJ0719, JXXY, WX, KLQ, KF, and PG45, respectively.

Supplementary Table 1 Information on the predicted proteins in the *Mycoplasma bovis* HB0801 genome.

Supplementary Table 2 Information on all proteins identified by the label-free quantitative proteomic technique.

Supplementary Table 3 Proteins with differential abundance between *Mycoplasma bovis* strains P1 (virulent) and P150 (attenuated).

Supplementary Table 4 Results of the computational prediction and proteomic analysis for the final secretomes of the *Mycoplasma bovis* P1 (virulent) and P150 (attenuated) strains.

Supplementary Table 5 Gene ontology enrichment analysis for overlapped proteins between the computational prediction and proteomic analysis.

Supplementary Table 6 The specificity of rMbovP0145-iELISA.
