# Supplementary figures and images for: Comparative Secretome Analyses of Mycoplasma bovis Virulent and Attenuated Strains Revealed MbovP0145 as a Promising Diagnostic Biomarker
Source: Front Vet Sci. 2021 Jun 18;8:666769. doi: 10.3389/fvets.2021.666769 (PMC8249566; doi:10.3389/fvets.2021.666769)

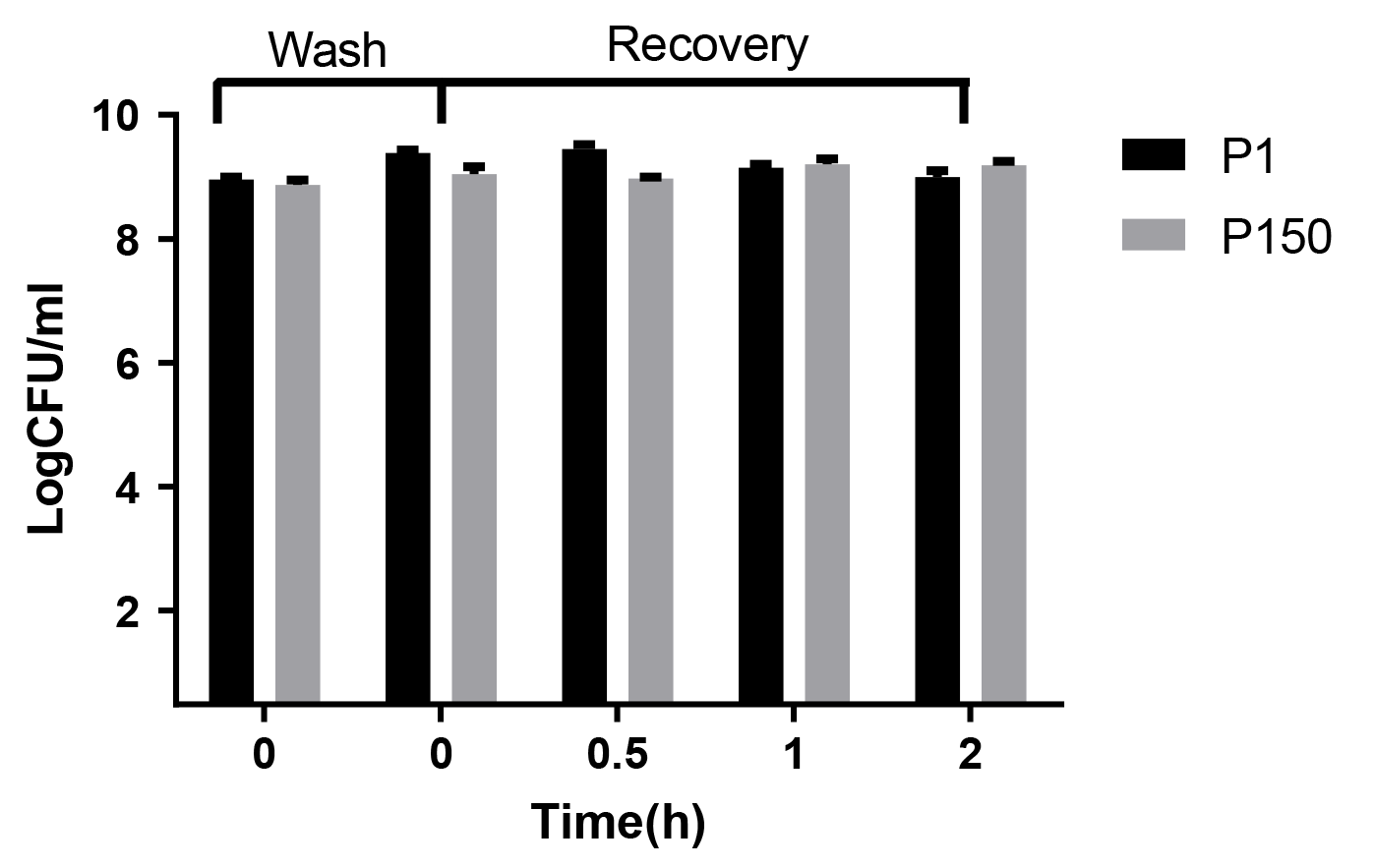

Supplement: Supplementary Figure 1 — Kinetic measurement of M. bovis growth during phosphate-buffered saline incubation. [file Data_Sheet_1.ZIP › Supplementary materials/Figure S1.tif]

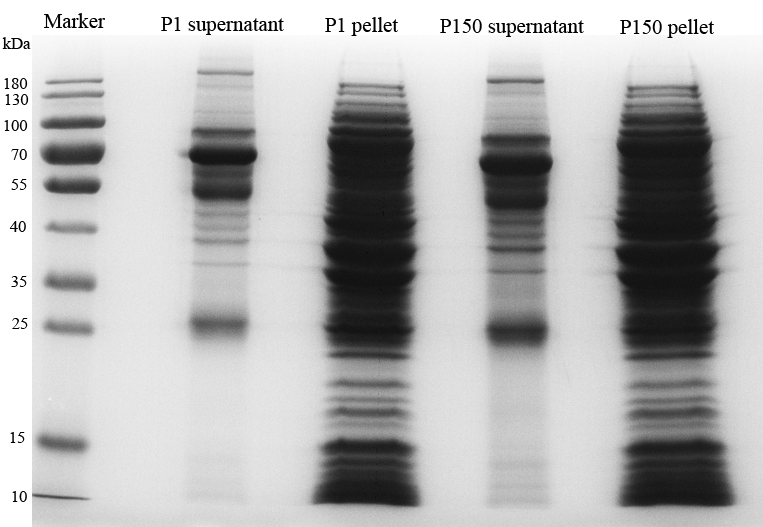

Supplement: Supplementary Figure 1 — Kinetic measurement of M. bovis growth during phosphate-buffered saline incubation. [file Data_Sheet_1.ZIP › Supplementary materials/Figure S2.tif]

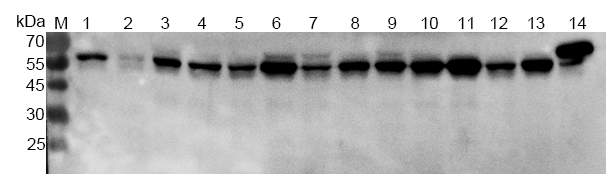

Supplement: Supplementary Figure 1 — Kinetic measurement of M. bovis growth during phosphate-buffered saline incubation. [file Data_Sheet_1.ZIP › Supplementary materials/Figure S4.tif]
